# Evaluation of fluorimetry and direct visualization to interpret results of a loop-mediated isothermal amplification kit to detect *Leishmania* DNA

**DOI:** 10.1186/s13071-018-2836-2

**Published:** 2018-04-17

**Authors:** Ana V. Ibarra-Meneses, Israel Cruz, Carmen Chicharro, Carmen Sánchez, Sylvain Biéler, Tobias Broger, Javier Moreno, Eugenia Carrillo

**Affiliations:** 1 WHO Collaborating Centre for Leishmaniasis, National Centre for Microbiology, Instituto de Salud Carlos III, Madrid, Spain; 2 Foundation for Innovative New Diagnostics, Geneva, Switzerland

**Keywords:** Leishmaniasis, Visceral leishmaniasis, Diagnostics, Loop-mediated isothermal amplification, LAMP, Less-invasive diagnosis, Non-invasive diagnosis, Loopamp™ *Leishmania* detection kit, Real-time fluorimeters, Boil & Spin

## Abstract

**Background:**

Nucleic acid amplification tests (NAATs) have proven to be advantageous in the diagnosis of leishmaniases, allowing sensitive diagnosis of: (i) cutaneous leishmaniasis in long duration lesions and (ii) visceral leishmaniasis using a less-invasive sample like peripheral blood, in opposition to tissue aspiration required for parasite demonstration by microscopy. Despite their benefits, the implementation of NAATs for leishmaniasis diagnosis at the point-of-care has not been achieved yet, mostly due to the complexity and logistical issues associated with PCR-based methods.

**Methods:**

In this work, we have evaluated the performance of a ready-to-use loop-mediated isothermal amplification (LAMP) kit using two real time fluorimeters to amplify leishmanial DNA obtained by silica column-based and Boil & Spin protocols.

**Results:**

The different approaches used to run and interpret the LAMP reactions showed a performance equivalent to PCR and real-time PCR, using spiked and clinical samples. The time to positivity obtained with real-time fluorimetry showed an excellent correlation with both *Ct* values and parasite load from real-time quantitative PCR.

**Conclusions:**

The results obtained open the possibility of using a highly stable, ready-to-use LAMP kit for the accurate diagnosis of leishmaniasis at the point-of-care. Furthermore, the feasibility of relating time to positivity, determined with a portable real-time fluorimeter, with the parasite burden could have a wider application in the management of leishmaniasis, such as in treatment efficacy monitoring or as a pharmacodynamics tool in clinical trials.

## Background

The loop-mediated isothermal amplification (LAMP) method enables the robust, fast, simple and highly specific amplification of nucleic acids. Since it was developed, LAMP has been used in several molecular diagnostic applications [1, 2]. In the field of infectious diseases it has made possible the diagnosis at the point-of-care (POC) level. For example, LAMP kits have been successfully developed for malaria and tuberculosis by the Eiken Chemical Co. (Japan), and the latter was recently endorsed by the World Health Organization [3–5]. The development of simple and easy-to-implement nucleic acid amplification tests (NAATs) such as LAMP has gained attention as these methods could enable accurate diagnosis at the POC in developing countries where access to laboratories is limited [6].

A LAMP kit for the diagnosis of leishmaniasis at the POC, the Loopamp™ *Leishmania* Detection Kit, has been recently developed by the Eiken Chemical Co., FIND and partners. This LAMP test uses primers targeting the *18S* rRNA gene and the kDNA minicircles, and is specific for the genus *Leishmania*. The kit is in a ready-to-use format and is composed of dried-down reagents, including primers, *Bacillus stearothermophilus* (*Bst*) DNA polymerase and calcein, so that no cold chain for transport and storage is required, and reactions can be set up at ambient temperature. The kit allows for different approaches to detect the amplified products. Before amplification, calcein contained in the dried reagents in each of the reaction tubes is in a quenched state, bound to manganese ions. Once the LAMP reaction starts, the pyrophosphate ions that are generated bind to the manganese ions, releasing calcein, thus generating fluorescent light that is detectable by the naked eye. This signal can be enhanced by illumination with UV or blue light. Real-time monitoring of the fluorescence associated to the amplification is possible using a fluorimeter, and it is also possible to assess the amplification using a real-time turbidimeter to measure the turbidity caused by the precipitation of magnesium pyrophosphate, a by-product of the reaction.

LAMP can amplify target DNA in different biological matrices and even in the presence of fresh blood components that usually inhibit PCR reactions, such as haemoglobin, IgG or IgM [7, 8]. However, there can be situations where the visual assessment of the amplified products can be challenging, like in crude DNA lysate preparations from blood [9, 10]. In addition, direct examination of changes in fluorescence intensity with the naked eye, even under UV or blue light, is dependent on, often subjective, human interpretation. To overcome these problems a number of approaches have been developed during the last decade to improve the detection of the amplified products either by end-point or real-time assessment, including electrochemical, optical and pH-sensing-based detection systems [8, 11]. Various incubators that can be used to run LAMP reactions are commercially available. Some of them, such as the LF-160 (Eiken Chemical Co., Japan), are designed for visual assessment of test results by naked eye using a blue light. Others, such as the LA-320C (Eiken Chemical Co.) can perform real-time turbidity measures. Both the LF-160 and the LA-320C are designed to accommodate LAMP reaction tubes made by Eiken Chemical, whose shape is slightly different from standard PCR tubes. By contrast, incubators made by other manufacturers are usually not designed for Eiken Chemical tubes. For example, the Genie III® (OptiGene Limited, Horsham, UK) is a compact, portable, battery-operated incubator including real-time fluorimetry, which is designed to accommodate standard PCR tubes. In 2014, FIND facilitated a modification of the design of the Genie III® by OptiGene so that it could be used with LAMP tubes made by Eiken Chemical. FIND also supported a study at the Institute of Primate Research (Kenya) to compare the LF-160, the LA-320C and the Genie III® using the Loopamp™ *Trypanosoma brucei* Detection Kit produced by Eiken Chemical (manuscript in preparation). To contribute to this subject we evaluated the performance of the Loopamp™ *Leishmania* Detection Kit using two different real-time fluorimeters and an incubator with coupled blue LED light illumination.

## Methods

### Study site

The study was conducted at the WHO Collaborating Centre for Leishmaniasis, National Centre for Microbiology, Instituto de Salud Carlos III, Madrid, Spain (WHOCCL-ISCIII), which is also the national reference laboratory for leishmaniasis.

### Biological materials

The following biological materials were used to create the panels tested in the three experiments described below: (i) *Leishmania infantum* promastigotes (strain MHOM/ES/2014/LLM-2240) cultured in Novy-MacNeal-Nicolle medium medium; (ii) heparin-treated peripheral blood and bone marrow samples from confirmed and non-confirmed visceral leishmaniasis (VL) suspects stored at -20 °C at the WHOCCL-ISCIII collection on leishmaniasis, registered at the National Biobank Register, Section Collections, Spain with the collection Reference ID: C.0000898; and (iii) freshly collected heparin-treated peripheral blood from a healthy volunteer.

### DNA extraction procedures

DNA was purified from pellets of cultured *L. infantum* promatigotes and from 95 μl of heparin-treated peripheral blood and bone marrow aspirates using the QIAamp DNA Mini Kit (Qiagen, Hilden, Germany). The DNA was eluted in 100 μl PCR grade water and processed immediately or stored at -20 °C until analysis. When DNA was extracted from *L. infantum* promastigotes its concentration was estimated using a NanoDrop™ spectrophotometer (Thermo Scientific, Wilmington, USA) and adjusted according to the experiments described below. Measures were taken in duplicates. Additionally, DNA was also purified from biological samples using a “Boil & Spin” protocol, as follows: 95 μl heparin-treated whole blood or bone marrow aspirate was mixed with 5 μl 10% SDS by inversion 10 times in a 1.5 ml microcentrifuge tube with screw cap, allowed to stand for 10 min at room temperature and mixed again. After adding 400 μl PCR grade water the mix was incubated in a heating block at 90 °C for 10 min. The mix was then spun for 3 min at maximum speed in a bench top microcentrifuge (13,000× *rpm*) and the supernatant containing the DNA was recovered and processed immediately or stored at -20 °C until analysis.

### Nucleic acid amplification tests

#### LAMP

LAMP reactions using the Loopamp™ *Leishmania* Detection Kit (Eiken Chemical Co.) were performed using 3 μl purified DNA and run in two different fluorimeters and one incubator, as described below.

#### Nested PCR and real-time quantitative PCR

DNA samples were also tested by *Leishmania* nested-PCR (LnPCR) and real-time quantitative PCR (qPCR), both targeting the *18S* rRNA (*SSU* rRNA) gene. Biological samples from the WHOCCL-ISCIII collection had been previously tested by LnPCR, with VL confirmed cases being positive, and non-confirmed VL suspects and healthy controls being negative. This test was repeated in this study to confirm sample integrity. LnPCR was performed with 3 μl DNA following the procedure described elsewhere [12]. qPCR was also performed with 3 μl DNA using a LightCycler 2.0 high speed thermal cycler and the LightCycler FastStart DNA Master SYBR Green I kit (Roche Diagnostics, Mannhein, Germany) following the protocol described elsewhere. Quantification was accomplished by means of a standard curve which consisted of serial dilutions of *L. infantum* DNA (strain JPC, MCAN/ES/98/LLM-722) ranging from the equivalent of 10^4^ to 10^-7^ parasites/μl [13, 14].

All LAMP, LnPCR and qPCR runs included positive (DNA equivalent to 10^4^ parasites/μl) and negative controls (DNA from peripheral blood from a healthy volunteer). LAMP, LnPCR and qPCR reactions were run in duplicate.

### Incubators/fluorimeters and interpretation of LAMP results

Loopamp™ *Leishmania* Detection Kit reactions were carried out in three different devices that allowed for the isothermal incubation required for the LAMP reaction. LAMP reactions were set up in all devices to run for 40 min at 65 °C. A final step of 80 °C for 5 min was applied to inactivate the *Bst* DNA polymerase.

#### LF-160 incubator

LF-160 incubator (Eiken Chemical Co.), now commercialized as HumaLoop M incubator (HUMAN, Wiesbaden, Germany), is a benchtop device for isothermal amplification. Results were visualized under blue LED light illumination, using the Fluorescence Visual Check Unit integral to the incubator [15]. Positive samples emit a green fluorescent light. Negative samples do not emit any light.

#### Genie III® real-time fluorimeter

The Genie III® (OptiGene Ltd.) is a hand-held, battery operated, device for isothermal amplification and real-time fluorescence detection at the point-of-care. Further information can be found elsewhere [16]. The Genie III® heat block was customized to match the dimension of the Loopamp™ *Leishmania* detection tubes and prevent tubes from opening during incubation. To allow for the measurement of the LAMP fluorescence, the Genie III® was set up for 470 nm excitation and 510–560 nm on the detector side. Prior to using the device for sample measurements, the gain of the detector was adjusted using positive and negative control wells from the Loopamp™ kit.

#### ESEQuant TS2.2 real-time fluorimeter

The ESEQuant TS2.2 (Qiagen Lake Constance GmbH) is a benchtop device for isothermal amplification and real-time fluorescence detection. Further information can be found elsewhere [17]. Fluorescence was monitored setting the dual fluorescence channel to 470 nm and 520 nm for excitation and emission, respectively. Prior to using the device for sample measurements, the gain of the detector was adjusted using positive and negative control wells from the Loopamp™ kit.

When using the fluorimeters, positive or negative results, as well as amplification time to positivity (*Tp*) in the Genie III®, were recorded upon examination of the amplification curves.

### Serological and parasitological tests in samples from VL suspects and healthy controls

Before their inclusion in the WHOCCL-ISCIII collection, samples were characterized, when applicable, by serological and parasitological methods as follows. All the samples from non-confirmed VL suspects and healthy negative controls had a negative result by both the serological and parasitological methods described below.

#### Immunofluorescent antibody test (IFAT)

Plasma samples (1 μl) from VL suspects and healthy controls were tested by an in-house IFAT following a standard method [18]. In this method the antigen is prepared from *L. infantum* promastigotes (strain MHOM/FR/78/LEM-75) and antibody binding is detected using fluorescein isothiocyanate-conjugated sheep anti-human immunoglobulin G (heavy and light chains). The threshold titre for positivity is ≥ 1/80.

#### Parasitology in bone marrow aspirates from VL suspects

Bone marrow aspirates were also used to prepare Giemsa smears and/or inoculate NNN cultures. Slides were examined at 1000× magnification for at least 1 h. NNN cultures were maintained at 27 °C, sub-cultured and examined by light microscopy on a weekly basis for at least 4 weeks before a negative result was returned.

### Panel of experiments

The following experiments were conducted to evaluate the performance of Loopamp^TM^ *Leishmania* Detection Kit using different methods to run the LAMP reaction and read the results.

#### Experiment 1

Ten-fold serial dilutions ranging from 10^3^ to 10^-7^ parasite equivalents/μl were prepared with DNA obtained from *L. infantum* promastigotes with the QIAamp DNA Mini Kit. We assumed 200 fg DNA as the equivalent to one parasite [13]. Three μl of each dilution were used in the LAMP, LnPCR and qPCR reactions in this and the other two experiments described below.

#### Experiment 2

*Leishmania infantum* promastigotes from stationary phase culture were counted in a Neubauer chamber, their number adjusted and mixed with heparin-treated peripheral blood from healthy negative control to prepare serial 10-fold dilutions of 200 μl each, ranging from 10^3^ to 10^-2^ parasites/μl. Ninety-five microliters was used in each DNA extraction method described above (QIAamp DNA Mini Kit and Boil & Spin).

#### Experiment 3

DNA was obtained by Boil & Spin and the QIAamp DNA Mini Kit from 95 μl aliquots of five heparin-treated human peripheral blood samples and five bone marrow aspirate samples from patients with suspected VL, which had been received at the WHOCCL-ISCIII between September and October 2015 and stored in their collection at -20 °C. These VL suspects were classified as confirmed or non-confirmed cases based on previous LnPCR, IFAT and parasitology results.

### Correlation analysis

In Experiment 1 we recorded data on time to positivity (*Tp*) for LAMP, obtained with the Genie III® fluorimeter, and qPCR cycle threshold (*Ct*) and parasite load. We estimated the Spearman’s correlation coefficient between *Tp* and both *Ct* and parasite load. These analyses were performed with SPSS software (IBM SPSS Statistic 22) and GraphPad Prism v.7.0 (GraphPad Software, San Diego, USA).

## Results

**Fig. 1:**
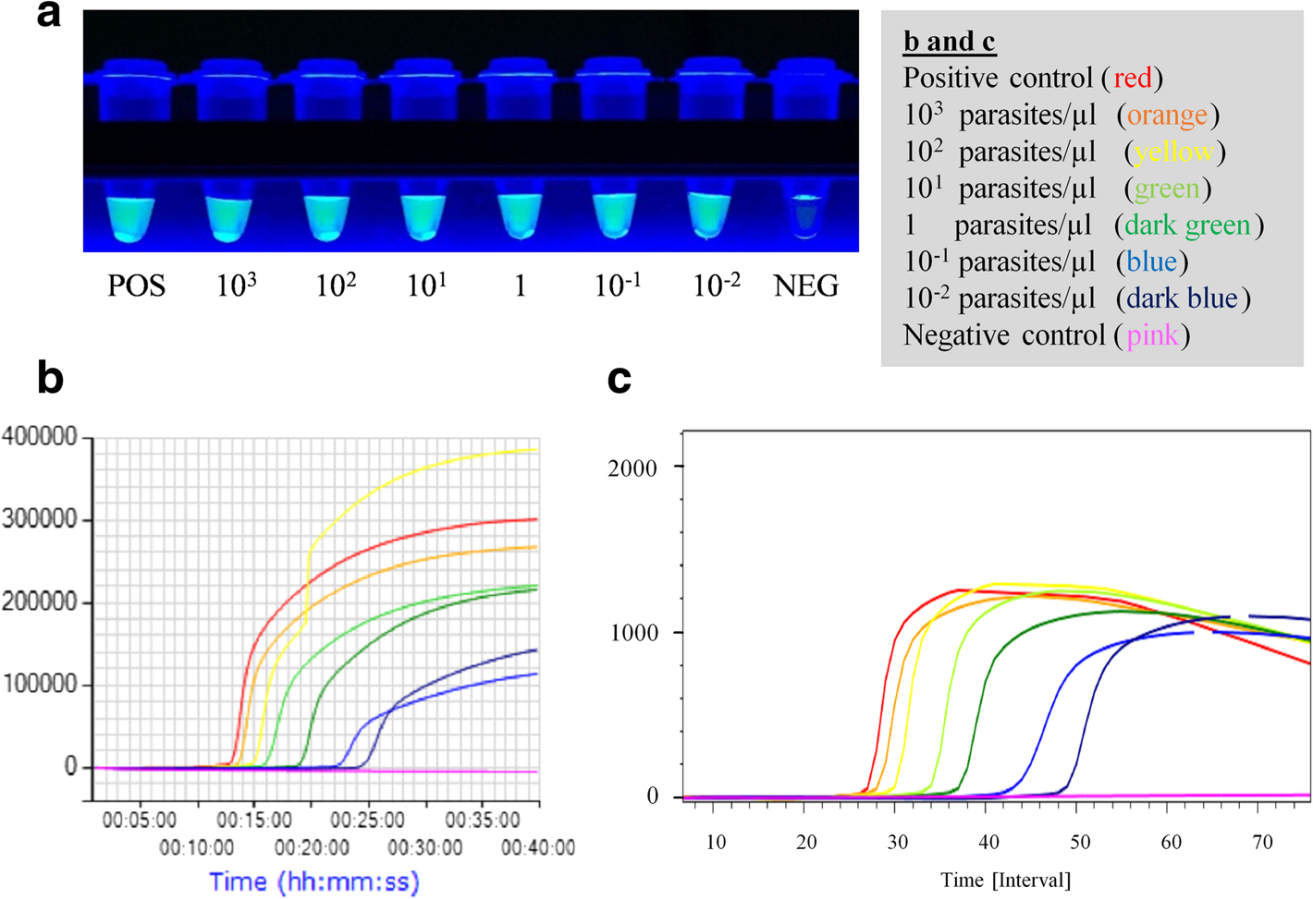
LAMP reaction results in Experiment 2 with the three different approaches proposed in this study. **a** Visual inspection under blue LED light illumination (Incubator LF-160) (POS, positive control; NEG, negative control; 10^3^ to 10^-2^ parasites/μl equivalents). **b** Amplification curves obtained using the real-time fluorimeter Genie III. Fluorescence in millivolts (mV) on the y-axis *vs* time (min) on the x-axis. **c** Amplification curves obtained using the real-time fluorimeter ESE-Quant TS2.2. Fluorescence in millivolts (mV) on the y-axis (×100) *vs* time (min) on the x-axis

The identification of positive results by examination of the amplification curves obtained with the Genie III® and ESEQuant TS2.2 fluorimeters was quite straightforward. Likewise, visualization under blue LED light illumination with th*e* LF-160 incubator allowed easy detection of positive (green) and negative (colourless) samples (Fig. 1).

**Fig. 2:**
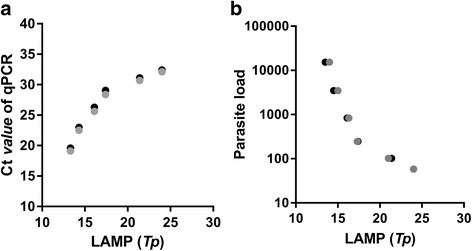
Correlation between LAMP and qPCR in the same set of samples (10^3^ to 10^-2^ parasites/μl), from Experiment 1. **a** Correlation between the *Tp* obtained with the Genie III® fluorimeter and the *Ct* values from qPCR (Spearman’s *r* = 0.9895, *P* = 0.0001). **b** Correlation between the *Tp* obtained with the Genie III® fluorimeter and parasite load from qPCR (Spearman’s *r* = -0.9912, *P* = 0.0001)

Independently of the device used for isothermal amplification and detection, LAMP detected all *L. infantum* DNA samples in the range prepared (10^3^–10^-3^ parasite equivalents/μl) in Experiment 1, while the limit of detection for LnPCR and qPCR was 10^-2^ parasite equivalents/μl (Table 1). We also observed a very strong correlation between the *Tp* obtained with the Genie III® fluorimeter and the *Ct* values from qPCR in the same set of samples (Spearman’s *r* = 0.9895, *P* < 0.0001, 95% CI: 0.9601–0.9972). Indeed, we also found a very strong correlation between the *Tp* obtained with the Genie III® fluorimeter and the parasite load determined by qPCR (Spearman’s *r* = -0.9912, *P* < 0.0001, 95% CI: -0.9977– -0.9666) (Fig. 2).

**Table 1:** Results obtained by using DNA isolated with QIAgen columns with the different instruments in Experiment 1

Instrument	*L. infantum* 10-fold DNA dilutions of parasite equivalents/μl										
	10^3^	10^2^	10	1	10^-1^	10^-2^	10^-3^	10^-4^	10^-5^	10^-6^	10^-7^
LF-160	Pos	Pos	Pos	Pos	Pos	Pos	Pos	Neg	Neg	Neg	Neg
Genie III®	Pos	Pos	Pos	Pos	Pos	Pos	Pos	Neg	Neg	Neg	Neg
*Tp* (min:sec)	13:30	14:30	16:15	17:45	21:45	24:00	34:15	0	0	0	0
ESEQuant TS2.2	Pos	Pos	Pos	Pos	Pos	Pos	Pos	Neg	Neg	Neg	Neg
LnPCR	Pos	Pos	Pos	Pos	Pos	Pos	Neg	Neg	Neg	Neg	Neg
qPCR	Pos	Pos	Pos	Pos	Pos	Pos	Neg	Neg	Neg	Neg	Neg
Ct	19.1	22.5	25.6	28.4	30.7	32.1	Neg	Neg	Neg	Neg	Neg

*Abbreviations*: *Pos* positive, *Neg* negative

In Experiment 2 (serial 10-fold dilutions of *L. infantum* promastigotes in heparin-treated peripheral blood), all molecular tests and approaches used detected *L. infantum* spiked samples in the range of 10^3^ to 10^-2^ parasites/μl, independently of the method used for DNA extraction (QIAamp DNA Mini Kit or Boil & Spin).

Experiment 3 showed a complete agreement between LnPCR, qPCR and LAMP when it was run in the real-time fluorimeters, independently of the DNA extraction procedure used, as well as in the LF-160 incubator when DNA samples were prepared with the QIAamp DNA Mini Kit. However, when samples prepared with the Boil & Spin method were run in the LF-160 incubator, the examination under blue LED light returned three discordant results even after repeated testing (Table 2). It was not possible to get a clear result for a bone marrow aspirate from a VL confirmed patient (VL6), and two samples from non-confirmed VL suspects (VL7-peripheral blood and VL8-bone marrow) returned a signal that was judged as positive when all other tests were negative (LnPCR, qPCR and LAMP as well as serology and parasitology).

**Table 2:** Results obtained with the different instruments in each of the different experiments proposed. Discordant results are shown in bold letters. Sample order in the table is the same as in LAMP analyses

Sample ID/ patient type	Sample type	LnPCR	qPCR	LAMP results by instrument/DNA preparation					
				LF-160 /B&S	LF-160 /QIA	Genie III® /B&S	Genie III® /QIA	ESEQuant TS2.2/B&S	ESEQuant TS2.2/QIA
VL1/C	Peripheral blood	Pos	Pos	Pos	Pos	Pos	Pos	Pos	Pos
VL2/C	Bone marrow aspirate	Pos	Pos	Pos	Pos	Pos	Pos	Pos	Pos
VL3/C	Peripheral blood	Pos	Pos	Pos	Pos	Pos	Pos	Pos	Pos
VL4/C	Peripheral blood	Pos	Pos	Pos	Pos	Pos	Pos	Pos	Pos
VL5/NC	Bone marrow aspirate	Neg	Neg	Neg	Neg	Neg	Neg	Neg	Neg
VL6/C	Bone marrow aspirate	Pos	Pos	**Unc**	Pos	Pos	Pos	Pos	Pos
VL7/NC	Peripheral blood	Neg	Neg	**Pos**	Neg	Neg	Neg	Neg	Neg
VL8/NC	Bone marrow aspirate	Neg	Neg	**Pos**	Neg	Neg	Neg	Neg	Neg
VL9/C	Bone marrow aspirate	Pos	Pos	Pos	Pos	Pos	Pos	Pos	Pos
VL10/NC	Peripheral blood	Neg	Neg	Neg	Neg	Neg	Neg	Neg	Neg

*Abbreviations*: *ID* Identification number in this study, *Pos* Positive result, *Neg* negative result, *C* confirmed VL case, *NC* non-confirmed VL case, *Unc* uncertain result

## Discussion

Leishmaniasis is a neglected tropical disease that has its largest impact on the poorest of the poor in endemic regions, which quite often do not have access to an accurate diagnosis due to a limited health service infrastructure. Prompt diagnosis and treatment is key to reduce the morbidity and stigma associated with cutaneous leishmaniasis (CL) and to avoid the chronic wasting and death of those affected by visceral leishmaniasis (VL) [19]. NAATs (principally PCR-based methods) have proven to be advantageous in the diagnosis of leishmaniasis, allowing the accurate diagnosis of CL even in lesions of long duration. The latter has enabled confirmatory diagnosis of VL using peripheral blood, in opposition to more invasive procedures such as bone marrow or splenic aspiration required for microscopy [20–23]. The diagnosis and management of leishmaniasis would benefit from the implementation of accurate NAATs at the POC.

LAMP is a simple and robust NAAT, and different studies have shown a number of applications of prototype LAMP tests in the field of leishmaniasis. Several prototypes have shown to be useful in detecting *Leishmania* infection in sand flies, diagnosis of canine leishmaniasis, human VL, CL and post-kala-azar dermal leishmaniasis. In general, these studies have reported high sensitivity and specificity assessing LAMP results either using direct examination with the naked eye [24–32], or fluorimetry or turbidimetry [33–35]. Regarding fluorimetry, both Genie III® and ESEQuant TS2.2 devices have been used in the diagnosis of different pathogens using LAMP [36–41], to the best of our knowledge this is the first time these approaches are used to detect *Leishmania* DNA using the Loopamp™ *Leishmania* Detection Kit.

Our results with the Loopamp™ *Leishmania* Detection Kit and different detection systems show a strong correlation with LnPCR and qPCR methods normally employed by the WHOCCL-ISCIII for the diagnosis of different forms of leishmaniasis as well as in epidemiological investigations [12–14, 42–49]. When using DNA purified with the QIAamp DNA Mini Kit LAMP returned a one-log higher analytical sensitivity (equivalent to 10^-3^ parasites/μl) than LnPCR or qPCR (10^-2^ parasites/μl), which might well be a reflection of the number of copies per *Leishmania* genome of the DNA targets in these NAATs (Experiment 1). It is worth noting that while LnPCR and qPCR target the *18S* rRNA gene (> 100 copies/genome), the Loopamp™ *Leishmania* Detection Kit targets both *18S* rRNA and kDNA minicirles (the later with > 1000 copies/genome) [50]. This high sensitivity has been demonstrated in the set of clinical samples from suspected VL cases included in this study.

When we used fresh blood spiked with *L. infantum* promastigotes (Experiment 2), the LAMP test showed a strong correlation with LnPCR and qPCR, independently of the method used for DNA preparation. It was only in clinical samples prepared with the Boil & Spin method (Experiment 3) that we found three discrepancies when the reactions were run in the LF-160 incubator and the results examined by the naked eye under blue LED light. This problem with naked eye examination of LAMP results from crude DNA lysates from blood has been reported elsewhere [9, 10]. As we did not see this problem in Experiment 2, we suggest this might be related to the use of fresh *versus* frozen stored samples, as the clinical samples used in Experiment 3 were collected in 2015 and kept at -20 °C since then. We can speculate that excess hemolysis in frozen samples affected the interpretation of LAMP results by the naked eye when DNA is prepared with the Boil & Spin method [51, 52].

The possibility of using a ready-to-use amplification kit with a simple sample preparation method (Boil & Spin) and a fluorimeter could be attractive for the diagnosis of visceral leishmaniasis in resource-limited health infrastructures. The accuracy of this approach for other clinical manifestations of leishmaniasis such as cutaneous leishmaniasis or post-kala-azar leishmaniasis should also be tested. In addition, the accurate detection of asymptomatic dogs acting as reservoir for zoonotic visceral leishmaniasis and the confirmation of canine visceral leishmaniasis cases in the field would be valuable for the control of leishmaniasis.

In summary, our study shows a good performance of the Loopamp™ *Leishmania* Detection Kit using different devices for amplification and interpretation of results, as well as different approaches for DNA preparation. The use of real-time fluorimeters presents the additional advantage of an objective assessment of the amplification, which is less affected by the background components present in crude DNA lysates obtained with the Boil & Spin method. Additional advantages of these fluorimeters is their portability and that they offer opportunities for diagnostic connectivity using Wi-Fi or Bluetooth® [16, 17]. The high correlation between LAMP *Tp* and both qPCR *Ct* values and parasite load also opens the possibility of exploring the use of real-time LAMP as a semi-quantitative test, as proposed by other authors [53–59]. Similarly, though not validated in this study, the use of real-time turbidimeters would also provide significant advantages in the interpretation of LAMP results [58, 59].

## Conclusions

The Loopamp™ *Leishmania* Detection Kit has shown a very good diagnostic performance in this small-scale study, and the possibility of using a simple sample preparation method (Boil & Spin) and a portable and robust real-time fluorimeter opens an avenue for the diagnosis of leishmaniasis at the POC, enabling treatment when confirmatory diagnosis is required. This test can support the diagnosis of VL in situations in which serological diagnosis is useless such as in VL relapses and test-of-cure, VL/HIV co-infection, and the diagnosis of CL cases that require confirmatory diagnosis to initiate systemic treatment, which often presents high toxicity. This promising approach could also be explored in veterinary public health for the diagnosis of canine leishmaniasis.

## Abbreviations

CL: cutaneous leishmaniasis
*Ct*: cycle threshold
FIND: Foundation for Innovative New Diagnostics
IFAT: immunofluorescence antibody test
kDNA: kinetoplast deoxyribonucleic acid
LAMP: loop-mediated isothermal amplification
LED: light-emitting diode
LnPCR: *Leishmania* nested-PCR
NAATs: nucleic acid amplification tests
PCR: polymerase chain reaction
POC: point-of-care
qPCR: real-time quantitative PCR
rRNA: ribosomal ribonucleic acid
*Tp*: amplification time to positivity
VL: visceral leishmaniasis
WHOCCL-ISCIII: WHO Collaborating Centre for Leishmaniasis, Instituto de Salud Carlos III, Spain
